# Risk factors of brucellosis seropositivity in Bactrian camels of Mongolia

**DOI:** 10.1186/s12917-018-1664-0

**Published:** 2018-11-13

**Authors:** Chimedtseren Bayasgalan, Tungalag Chultemdorj, Felix Roth, Jakob Zinsstag, Jan Hattendorf, Battsetseg Badmaa, Bayanzul Argamjav, Esther Schelling

**Affiliations:** 1 School of Veterinary Medicine, Mongolian University of Life Sciences, PO Box 17024, Zaisan, Ulaanbaatar, Mongolia; 2 Basel, Switzerland; 3 Swiss Tropical and Public Health Institute, PO Box, CH-4002 Basel, Switzerland; 4 University of Basel, Basel, Switzerland

**Keywords:** Bactrian camel, Brucellosis, Epidemiology, Mongolia, Seroprevalence, *Brucella* spp., Risk factors

## Abstract

**Background:**

More information on brucellosis epidemiology in Bactrian camels is needed due to their growing economic and livelihood importance for herders and renewed efforts in Mongolia to eliminate brucellosis through mass vaccination of ruminants excluding camels. Brucellosis prevalence in camels increased over the past two decades. Random multi-stage cluster surveys were done in the Eastern provinces of Dornod and Sukhbaatar in 2013 and 2014 and in the Southern & Western provinces of Dornogobi, Umnogobi and Khovd in 2014 and 2015. A total of 1822 camels, 1155 cattle, and 3023 small ruminant sera were collected and tested with the Rose Bengal Test. In addition, 195 vaginal swabs and 250 milk samples for bacteriological culture were taken from livestock with history of abortion.

**Results:**

The overall apparent seroprevalence in camels was 2.3% (95% confidence interval 1.6–3.3). The main risk factor for camel seropositivity was being in an Eastern province when compared to Southern & Western provinces (odds ratio 13.2, 95% CI 5.3–32.4). Camel seroprevalences were stable over the two consecutive survey years, despite introduction of ruminant vaccination: 5.7% (95% CI 3.1–10.2%) and 5.8% (3.3–10.1%) in Eastern provinces and 0.4% (0.2–1.2%) and 0.5% (0.1–2.0%) in Southern & Western provinces. We isolated *Brucella abortus* from camels and cattle. Camel seropositivity was associated to keeping cattle together with camels. Monitoring of vaccination campaigns showed that coverage in cattle was insufficient because animals could not be adequately restrained.

**Conclusions:**

The present study reveals that brucellosis is present with important seroprevalence in Mongolian camels and was endemic in Eastern provinces. Camel herd seropositivity was most closely associated to infection in cattle.

Longer term monitoring is needed to assess whether camel seroprevalance decreases with ongoing vaccination in Mongolia. This should be coupled with further confirmation on *Brucella* spp. isolates. To date, only *Brucella abortus* was isolated, but camels are also susceptible to *Brucella melitensis*. Clear verbal and written information on disease prevention in livestock and household members is important, particularly for remote camel herders who had only moderate knowledge on brucellosis epidemiology and preventive measures.

**Electronic supplementary material:**

The online version of this article (10.1186/s12917-018-1664-0) contains supplementary material, which is available to authorized users.

## Background

The Bactrian camel (two humped) and the dromedary (one humped Arabian camel) represent the old-world domesticated camel species and are closely related [1, 2]. The Bactrian camel inhabits cold deserts in the southern areas of Russia, Mongolia, East-Central Asia and China [3].

Camel husbandry in Mongolia is practiced primarily by pastoralists in the Gobi Desert. Camels produce milk, wool and meat and are also used for racing and, less commonly now, for transportation of people and goods. In 2014, it was estimated that there were 367,900 camels in Mongolia [4]. The camel population resides in close contact with cattle, sheep, goats and occasionally horses, particularly at watering places (wells, branch-water, ditch-water, rivers, and lakes) and during calving and wool shearing periods. Camels, unlike other domestic large animals, often travel up to 16 km daily in search of food [5]. They are less susceptible to some highly contagious livestock diseases, such as foot-and-mouth disease [6].

Brucellosis is a zoonosis caused by the intracellular, Gram negative bacteria of the genus *Brucella*. Sheep and goats are the main hosts for *Brucella melitensis*, while cattle are the main host for *Brucella abortus* and pigs are the main host for *Brucella suis*. These three species cause the majority of the disease burden in animals and are also the most important *Brucella* pathogens in people. However, other species (e.g. *Brucella canis*) are also potentially infectious to humans [7, 8].

Brucellosis is thought to be the most economically important zoonosis worldwide because it is endemic in many countries and impacts both human and livestock health [9–11]. Brucellosis is transmitted from animals to people often through consumption of unpasteurized milk and dairy products [12–15], but direct contact, particularly with livestock abortion material, is more important among livestock-keeping communities. The disease is rarely fatal in people but causes high morbidity in both animals and humans [16, 17].

Camels are susceptible to both *B. abortus* and *B. melitensis* [18–20]; however, camels are considered to be secondary hosts of *Brucella* spp. [3, 14]. Brucellosis was reported in camels as early as in 1931 by Solonitsiun in Russia [18, 21]. Since then, serological evidence of brucellosis has been reported from the most important camel-keeping countries [3, 18, 21–23]. Camels infected with brucellosis show fewer clinical signs than other livestock species, in particular less than domesticated cattle, sheep and goats [24]. This may be a reason why little information is available on epidemiology of brucellosis in camels and its impact on human health, notably in Mongolia [3, 25].

Brucellosis serological tests have rarely been validated for camels. Empirically, the Rose Bengal test is commonly used for diagnosis in camels and seems to give accurate results [24, 26].

Camels were included in mass screening surveys in Mongolia, but risk factors for exposure were not further evaluated. A screening survey in 2011 [27], which sampled between 6 and 3590 camel sera from each of the 22 Mongolian provinces, found a moderate correlation (Spearman’s rho = 0.26) between camel and cattle brucellosis seropositivity at district level; however, sheep were very weakly correlated while goats were not at all correlated (unpublished data). There is almost no information on which *Brucella* spp. cause seropositivity in Mongolian camels due to a lack of strain isolation and characterization. Past and current mass livestock vaccination campaigns in Mongolia did not include camels or horses. Older reports from veterinary laboratories indicated that the serological prevalence of brucellosis in camels in different Mongolian localities was increasing [28]. Notably, in 2010 a 3% seroprevalence in camels was found in a population-based survey in Sukhbaatar province [29].

Camels may be a reservoir for *Brucella* spp., and other livestock are at risk for reinfection when vaccination campaigns are discontinued because they are kept together. However, effective control of brucellosis could be achieved by establishing diagnostic and surveillance systems, by estimating the cost-benefits of control measures to guide policy makers, by rigorously implementing control programs, and by policies to connect human health and veterinary services at demographic, socioeconomic and political levels. Ruminant (Bovidae) mass vaccination was estimated to be highly cost effective for Mongolia [30]. In a mobile context, test and slaughter is hardly feasible. Instead, vaccination of cattle and small ruminants over several years is the viable control measure for mobile livestock husbandry systems, where there is also no feasible individual animal tracking system. The required vaccination coverage to interrupt transmission, in cattle (minimum 60% truly immunized animals) and in small ruminants (minimum 40%), must be monitored [31]. Post-vaccination campaign monitoring in cattle and small ruminants is now undertaken. However, the role of Bactrian camels in brucellosis epidemiology must be more clearly understood for successful elimination efforts in Mongolia, in particular, the ability of camels to maintain an own infection cycle and reintroduce brucellosis to domesticated Bovidae.

The objectives of this study were to contribute to understanding the epidemiology of camel brucellosis in Mongolia and to identify the *Brucella* species involved before and after implementation of vaccination campaigns in cattle and small ruminants. We tested the hypotheses that the seroprevalence of camel brucellosis is below 5% in Mongolia and the most important risk factor of camel seropositivity was herding together with cattle.

## Results

A total of 6000 serum samples (1822 camels, 1155 cattle, 1531 sheep, 1492 goats) were collected from 365 herds in five provinces over 3 years. In addition, 195 vaginal swabs (72 from camels, 51 from cattle, 29 from sheep, 43 from goats) and 250 milk samples (104 from camels, 68 from cattle, 46 from sheep, 32 from goats) were collected for bacteriological culture. In total, 310 out of the 365 herds sampled completed a questionnaire during the study, with 240 being completed at the first visit of a herd.

**Fig. 1:**
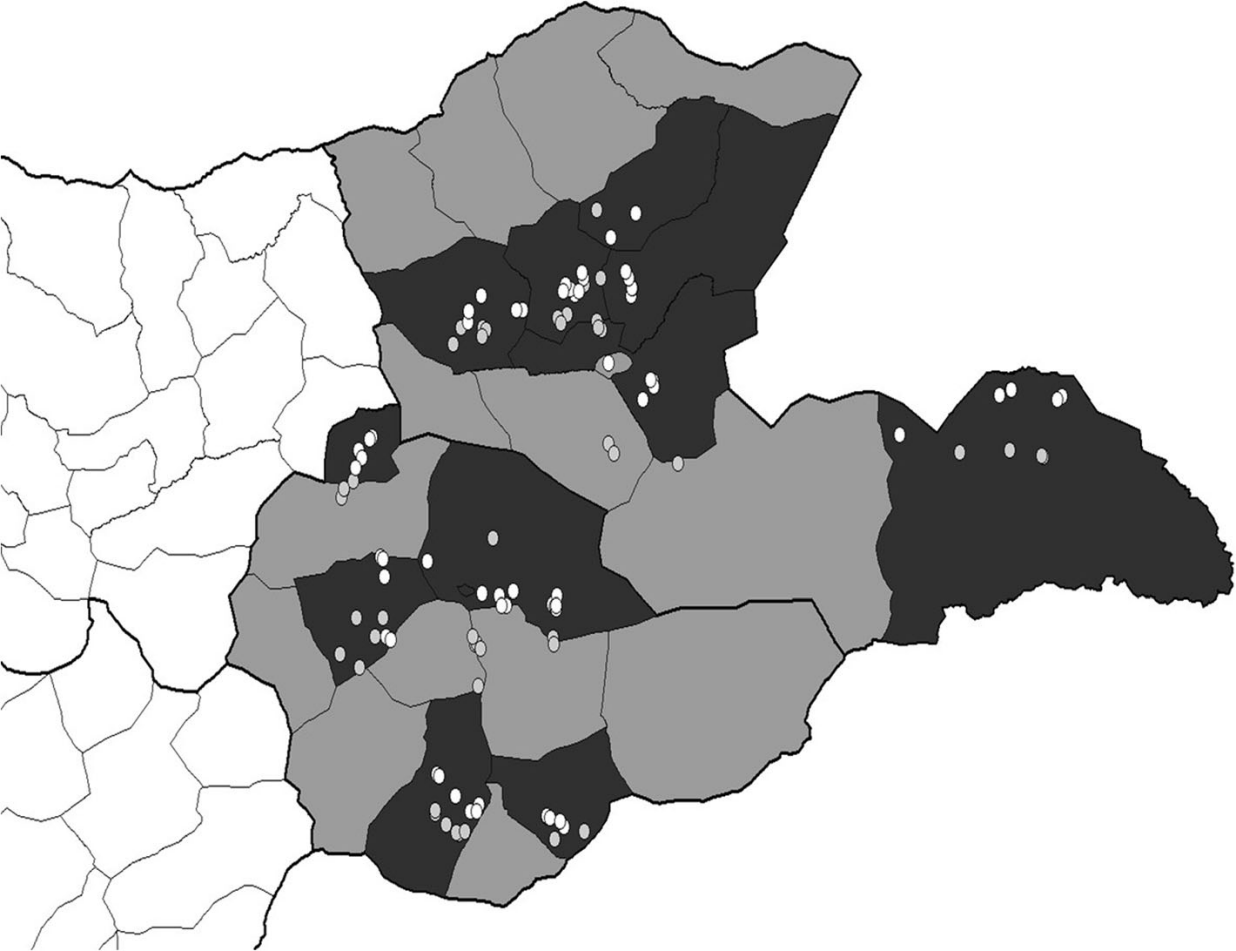
Map of Eastern provinces Dornod and Sukhbaatar (shaded in grey) and showing the selected districts (in black). The location of camel herds at time of sampling in 2013 (white dots) and 2014 (grey dots) are shown

**Fig. 2:**
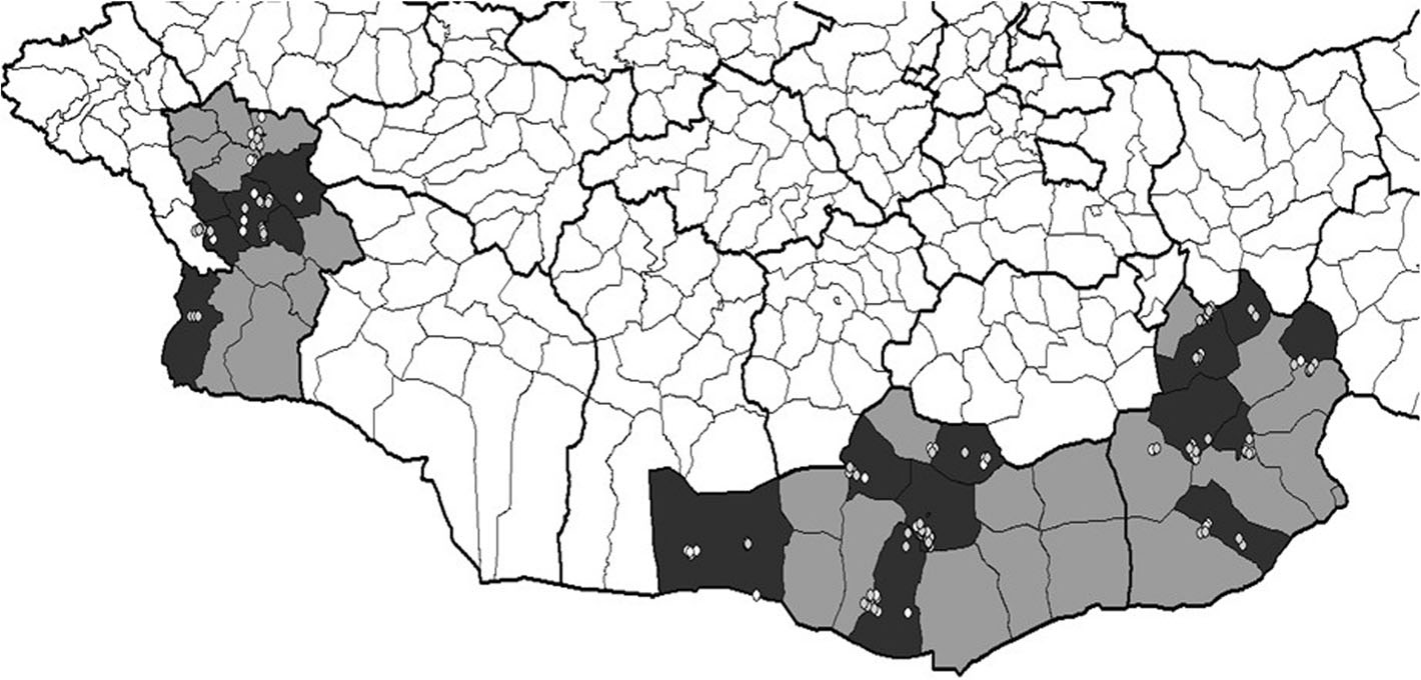
Map of Southern & Western provinces Dornogobi, Umnogobi and Khovd (shaded in grey) and showing the selected districts (in black). The location of camel herds at time of sampling in 2014 (white dots) and 2015 (grey dots) are shown. Due to movement of *hot ails* not all herds were subsequently found in the district where they were first registered

No camels were sampled in 9 herds, so the total camel herds was 356 (Table 1). The selected districts within the 5 provinces and the sites of sampling for the first and second years are depicted in Figs. 1 and 2.

**Table 1:** Distribution of the 356 camel herds sampled in 5 provinces over 3 years of sampling. In a second year the number of re-sampled herds and (+) the number of newly sampled herds is shown

	Year 1 (2013)	Year 2 (2014)	Year 3 (2015)
Dornod	32	24 + 8	
Sukhbaatar	37	34 + 4	
Dornogobi		36	22 + 14
Umnogobi		37	24 + 12
Khovd		36	19 + 17

A total of 1822 camel sera were tested, of which 17, 10 and 10 sera showed +, ++ and +++ postive agglutination, respectively. The 37 seropositive camels were in 29 of the 356 camel herds, and in herds with more than one positive camel different strengths of agglutination were seen. The overall apparent brucellosis seroprevalence in camels was 2.3% (95% CI 1.6–3.3). The estimated true seroprevalence was 1.8%. About one fifth of the camel sera collected originated from male animals. The majority of camels sampled were adults (13.9% young vs. 86.1% of adult camels), and seroprevalences were comparable across age groups (Table 2).

**Table 2:** Results of camel seroprevalences by the Rose Bengal Test (RBT) stratified by sex, age class, province and sampling year

Variable	Category	n	n pos	Seroprevalence^b^	95% CI^b^
Province	Dornod	241	13	5.3	2.9–9.6
	Sukhbaatar	298	18	6.1	3.5–10.6
	Dornogobi	388	3	0.8	0.3–2.3
	Umnogobi	526	2	0.4	0.1–1.4
	Khovd	369	1	0.3	0.04–1.9
Sex	Female	1429	26	2.2	1.4–3.4
	Males	332	10	3.0	1.6–5.5
Age class	≤ 4 years	253	5	2.0	0.8–4.7
	> 4 years	1569	32	2.3	1.6–3.4
Year	Eastern provinces 2013	237	13	5.7	3.1–10.2
	Eastern provinces 2014	302	18	5.8	3.3–10.1
	Southern & Western provinces 2014	897	4	0.4	0.2–1.2
	Southern & Western provinces 2015	386	2	0.5	0.1–2.0

^a^Positive with RBT, ^b^95% confidence interval (CI) calculated with the panel variable on the level of herd to consider potential clustering within herds; Eastern provinces: Sukhbaatar and Dornod Southern & Western provinces: Dornogobi, Umnogobi and Khovd

Camel brucellosis seropositivity was highest in Sukhbaatar (6.1%, 95% CI 3.5–10.1%) followed by Dornod (5.3%, 2.9.-9.6%), Dornogobi (0.8%, 0.3–2.3%), Umnogobi (0.4%, 0.1–1.4%), and Khovd (0.3%, 0.04–1.9%). The camel seroprevalences remained steady between the first and second years of sampling with 5.7% (95% CI 3.1–10.2%), and 5.8% (95% CI 3.3–10.1%) in Eastern provinces, and, at much lower levels, in the Southern & Western provinces with 0.4% (0.2–1.2%) in 2014 and 0.5% (0.1–2.0%) in 2015 (Table 2).

Regarding risk factors, camel age and sex were not significantly associated with seropositivity. The Eastern provinces had significantly higher seropositive proportions than the Southern & Western provinces (Table 3). Keeping camels together with cattle was significantly associated to brucellosis seropositivity in camels, whereas the presence of small ruminants was not. Out of all camels sampled, 86.9%, 93.1%, and 94.4% were kept together with cattle, sheep and goats, respectively (Table 3).

**Table 3:** Analysis of risk factors for camel seropositivity, multivariable analysis showing odds ratios using a generalized estimating equation (GEE) model considering the panel variable at herd level

		n neg	% neg	n pos	% pos	OR	95% CI	*p*-value
Province	Dornogobi	385	99.2	3	0.8	ref		
	Dornod	228	94.6	13	5.4	7.9	2.1–30.1	0.003
	Khovd	368	99.7	1	0.3	0.4	0.05–3.2	0.4
	Sukhbaatar	280	94.0	18	6.0	10.2	2.7–38.6	0.001
	Umnogobi	524	99.6	2	0.4	0.5	0.1–2.4	0.4
Age class	≤ 4 years	248	98.2	5	1.8	ref		
	> 4 years	1537	98.0	32	2.0	1.2	0.4–3.2	0.7
Sex	Female	1403	99.2	26	1.8	ref		
	Male	322	97.0	10	3.0	0.8	0.3–1.8	0.5
Year	2013	224	94.5	13	5.5	ref		
	2014	1177	98.2	22	1.8	1.0	0.4–2.4	1.0
	2015	384	99.5	2	0.5	1.0	0.2–5.6	1.0
Cattle present	no	238	100	0	0.0	ref		
	yes	1547	97.7	37	2.3	8.1	1.5-inf	0.01^Ɨ^
Sheep present	no	126	100	0	0.0	ref		
	yes	1659	97.8	37	2.2	4.0	0.7- inf	0.1 ^Ɨ^
Goats present	no	102	100	0	0.0	ref		
	yes	1683	97.8	37	2.2	3.2	0.6- inf	0.2 ^Ɨ^

^Ɨ^ -exact logistic regression, * *p* ≤ 0.05

We found no association between camel seropositivity and history of abortion or preventive biosafety measures such as destroying abortion material (Table 3). None of the biosafety measure (e.g., buying of live animals, safely disposing of abortion material) question outcomes were associated with seropositivity in camels, nor was the variable with distances of camel herds to the closest district centre (mean distance was 55 km).

Owners of seropositive camels had significantly more sources of information on brucellosis, indicating that they were informed about brucellosis in their herd. However, knowledge of herders on brucellosis can only be judged as moderate. From a total of 38 possible scores of the three knowledge themes with 19 questions, the median score achieved by participants was 23.

At the herd level, no significant correlations were found between camel and ruminant seroprevalences with the regression model using bootstrapping, regardless of considering all herds or considering only herds in provinces with no ruminant vaccination to ensure that seropositivity in ruminants was not a result of vaccination (even though there is little possibility of seropositivity persisting from previous vaccination) (Table 4). Goats in provinces and years with on vaccination were negatively correlated.

**Table 4:** Regression coefficients using bootstrap re-sampling technique for camel herd seropositivity and within herd seropositivity of cattle, sheep and goats (all herds) and only for herds in a province without vaccination (no vaccination), ^a^ significant negative correlation

		N herds	Intercept (95% confidence interval [CI])	Slope (95% CI)
Ruminants	All herds	348	0.02 (−0.01–0.03)	0.06 (−0.07–0.21)
	No vaccination	137	0.03 (0.01–0.05)	0.08 (−0.14–0.5)
Cattle	All herds	292	0.03 (0.01–0.04)	0.06 (−0.02–0.2)
	No vaccination	103	0.04 (0.01–0.06)	0.06 (−0.06–0.3)
Sheep	All herds	333	0.03 (0.02–0.04)	0.03 (− 0.04–0.1)
	No vaccination	127	0.03 (0.01–0.05)	0.1 (− 0.2–0.7)
Goats	All herds	341	0.03 (0.02–0.04)	−0.003 (− 0.07–0.1)
	No vaccination	133	0.03 (0.01–0.05)	−0.1 (− 0.27 - -0.04)^a^

Variances of camel serpositivity were higher at herd level than province and district levels (Table 5). The ICC was estimated at herd level. For the cluster sample size calculation we assumed an ICC of 0.1 at herd level. In the Eastern provinces, this was nearly the case; however, the ICC was much lower in the Southern & Western provinces, where there were rarely seropositive camels. Clustering in herds is higher than in provinces or districts, therefore, correlation within herds (as the ecological unit) was accounted for in the statistical analysis.

**Table 5:** The variances and intraclass correlation coefficients (ICC) of camel seropositivity at different levels. The greater the variances between herds compared to the overall total variance, the higher the ICC

Eastern provinces	2013	2014	Both years
Variance at herd level	2.1	2.0	1.2
Variance at district level	0.16	0.48	0.4
Variance at province level	< 0.01	< 0.01	< 0.01
Calculated ICC for camel herds	0.2	0.12	0.06
Southern & Western provinces	2014	2015	Both years
Variance at herd level	5.6	2.85	2.2
Variance at district level	2.0	1.3	1.1
Variance at province level	< 0.01	< 0.01	< 0.01
Calculated ICC for camel herds	< 0.001	0.04	< 0.001

**Fig. 3:**
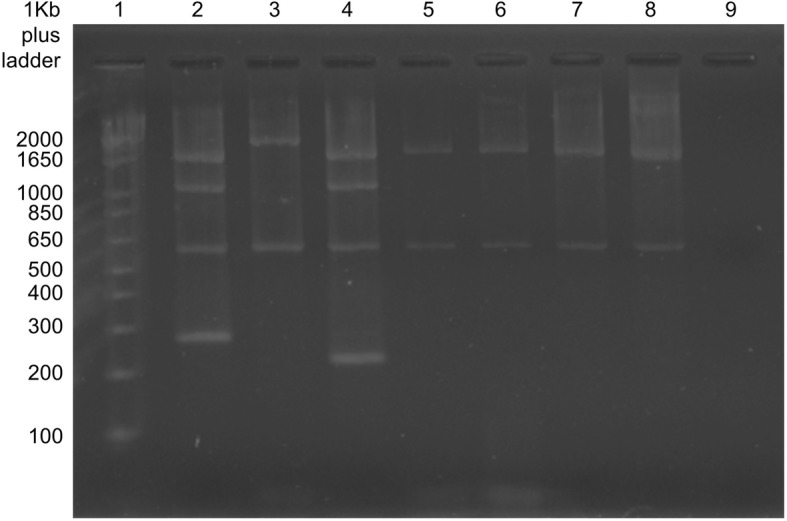
Agarose gel electrophoresis PCR products. Lane 1: DNA ladder; Lane 2: positive control *Brucella suis;* Lane 3: positive control *Brucella abortus* (vaccine strain RB51) with two bands at 2524 and 587 bp; Lane 4: positive control *Brucella melitensis* (vaccine strain Rev1); Lane 5: the isolate from a camel; Lanes 6–8: isolates from cattle; Lane 9: negative control

*Brucella* spp. were isolated from the milk of one camel and from three vaginal swabs of cattle. The four isolated *Brucella* strains were identified as *B. abortus* (Fig. 3).

## Discussion

A mass screening survey in all 22 provinces of Mongolia in 2011 reported seroprevalences of brucellosis in camels between 0.2 and 5.9% [28]. A previous (2010) population-based randomized survey in Sukhbaatar of Eastern Mongolia found a seroprevalence of 3% in camels [29]. In this study, we assessed seroprevalence and risk factors of camel seropositivity, in consideration of previous exposure to *Brucella* spp. There are shortcomings of using a serological test to define an outcome, as there will be false seronegative and false seropositive results, particularly when specificity of the test is low. Results subsequently need to be interpreted cautiously. In consideration of false positives, many authors set the cut-off for a seropositive herd as having at least two positive animals. In a complementary study on serological test characteristics comparing five different tests for use in camels, we concluded that the RBT is valid to assess brucellosis exposure status of Mongolian camels given its high specificity. However, due to lower sensitivity in camels when compared to other livestock species, we do not recommend it as a screening test for brucellosis monitoring in camels [26].

Between 2013 and 2015, seroprevalences in provinces showed high variation ranging from 0.3 to 6.1%, but the prevalences in regions were stable between the two sampling years. The ICC depends on the degree of clustering and also on the prevalence. The ICC used for the sample size calculation (0.1) was appropriate for the Eastern provinces; however, since a much lower ICC at herd level was calculated for the Southern & Western provinces, we may have slightly oversampled there [32, 33].

Brucellosis seroprevalences above 5% in livestock species are important, indicating endemic status [34]. Eastern provinces had significantly higher seroprevalences than Southern & Western provinces. Being in an Eastern province was the most important risk factor of camel brucellosis seropositivity, with an OR of 13.2 when compared to the Southern & Western provinces. The same result was seen when the cut-off value of camel seropositivity was set at higher agglutination (++ positivity). The majority of serological studies on brucellosis report higher seroprevalences in older animals [20, 35], which we did not see among camels. Nonetheless, another study reported that brucellosis infection began early in life, probably through suckling, and persisted into adulthood [14].

Public health education campaigns should continue among herders to inform them about brucellosis prevention practices and herd and human health management. Past surveys in the framework of monitoring vaccination outcomes coupled with human brucellosis prevalence found that all information sources (veterinarians, radio/TV, newsletters to herders, information brochures and newspapers) significantly improved herder knowledge on brucellosis epidemiology, prevention and clinical signs in both people and livestock. Since Mongolian herders are literate, both oral and written information material is appropriate.

Musa et al. [36] reported that cattle were a possible source of infection for camels because all small ruminants tested in their study were negative. Hadush et al. [20] reported that camel herds with close contact in pastures with cattle and small ruminants were 3.6 and 2.3 times, respectively, more at risk to be brucellosis seropositive than those with no contact. We found an association between camel seropositivity and cattle, but not small ruminants, keeping. The fact that our camel isolate was *B. abortus* further supports a linkage of brucellosis in cattle and in camels. This finding is consistent with the screening in all Mongolian provinces with a correlation of camel and cattle seropositivity at district level, as well as previous reports of identification on *Brucella* spp. from camels in Asia, where another isolate from a Mongolian camel also was *B. abortus* [37]. Monitoring surveys of achieved vaccination coverage from 2012 to 2015 indicate that sufficient coverage was achieved in small ruminants, but coverage was critically low in cattle. Veterinarians reported that cattle were difficult to restrain adequately to administer conjunctival vaccination. Achieving insufficient vaccination coverage in cattle in the first year of newly introduced ruminant vaccination campaigns could explain why camel seropositivity remained stable between the years, both without and with cattle vaccination.

## Conclusions

The results of this survey confirm the presence of *Brucella* spp. in camel herds in Mongolia. Camel seropositivity was significantly higher in Eastern than in Southern & Western provinces and was associated with keeping cattle together with camels. Decrease of camel brucellosis seropositivity was not observed despite ongoing ruminant vaccination. Repeated studies are needed to see if seroprevalences in camels drop over time with ongoing vaccination in other livestock species. Close attention should be given to achieve and monitor sufficient vaccination coverage in cattle in Mongolia. More isolates are needed to confirm that seropositivity in camels is limited to infection with *B. abortus*.

## Materials and methods

### Study design and selection of herds

**Fig. 4:**
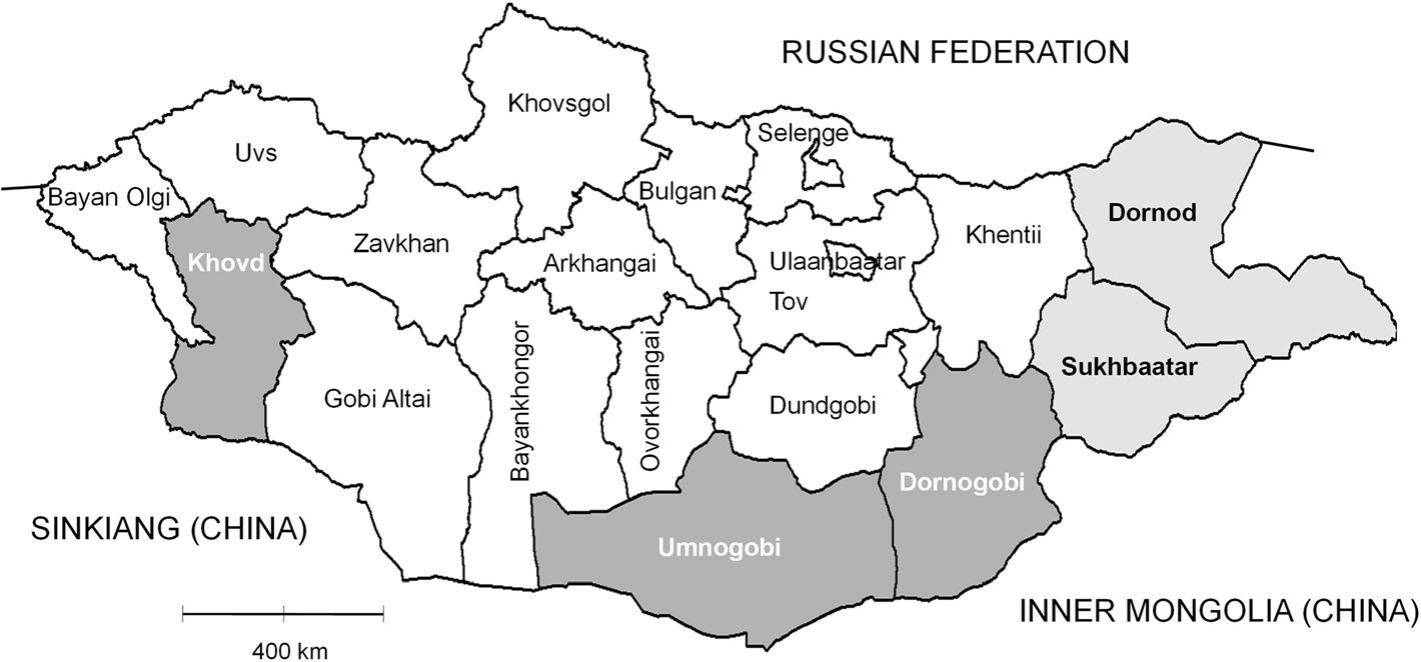
Map of Mongolia showing the surveyed provinces. The light grey provinces of Dornod and Sukhbaatar (Eastern provinces) were sampled in 2013, and a second survey was done in 2014. Surveys in the darker grey provinces of Khovd, Umnogobi and Dornogobi (Southern & Western provinces) started in 2014 and were repeated in 2015

We purposely selected the two Eastern provinces Sukhbaatar and Dornod for the first year of the study in 2013. The seroprevalence of brucellosis was high (> 3%) in camels in the multi-disease screening survey in Dornod in 2011 [27] and in Sukhbaatar during an epidemiological survey in 2010 [29]. Both provinces had a substantial number of camels and had not yet been included in the livestock (cattle, sheep and goats) brucellosis vaccination campaigns initiated in September 2013. Therefore, it was possible to sample before and after introduction of vaccination in 2014 in both Eastern provinces. The selection of three additional provinces in 2014 (second year of the study) was proportional to the size of their respective camel population as available from the annual livestock census [38]. Selection of provinces and districts proportional to size better ensured equal probability of camels to be enrolled in the study. The selected provinces had on average 32,500 camels per province. The Southern and Eastern provinces (Umnogobi, Dornogobi and Khovd) were surveyed in year 2 (2014) and year 3 (2015) (Fig. 4 and Table 1). During the study period, Umnogobi was the only province (out of 22) with no livestock brucellosis vaccination due to the large proportion of camels and vast size of the province. In areas using conjunctival vaccination of cattle and small ruminants, sampling was more than 5 months after vaccination campaigns, so the animals would no longer be seropositive due to vaccination [39, 40]. In each province, six districts were selected proportional to size of the camel population.

Repeated surveys using multi-stage cluster sampling were done in all provinces. Households with camels were randomly selected from lists of families registered with the district governor’s office. The epidemiological sampling unit in rural zones was the *hot ail*, typically 2–3 families which pasture their livestock together and share watering places during certain times of the year. The entire *hot ail* herd of a selected family was included. District veterinarians indicated the zone where a selected *hot ail* was at the time of sampling, then the study team would travel to the zone and ask encountered herders about precise locations for the selected *hot ail*. Six and 30 % of initially selected *hot ails* could not be sampled in Eastern and Southern & Western provinces, respectively. Reasons for non-participation were family moved too far away, family was preparing to move and did not have time or family’s camel herd was located too far from the *hot ail*. In such cases of non-participation, a replacement *hot ail* was enrolled which was either additionally selected from the district family list (the initial selection assumed that not all families would be found in a district) or from the nearest *hot ail* located in a northern direction from where the team determined that a selected *hot ail* could not be enrolled. For second year sampling in the same province, herders selected the previous year were contacted by mobile phone to establish their location and schedule the sampling. Reasons for non-participation were the same as for the first enrollment, and revisits were not possible in 10% and 40% in Eastern and Southern & Western provinces, respectively, so replacements were enrolled (Table 1).

#### Sample size

The sample size calculation assumed an intraclass correlation coefficient (ICC) of 0.1 for all livestock species. The ICC is the ratio of the variance between clusters over the total variance [41]. An ICC of 0.1 was reported for a range of endemic zoonoses [42] and was assumed, based on previous livestock brucellosis serological surveys in Mongolia [43]. This led to a design effect D of 1.2 and 1.4, when 3 and 5 animals, respectively, were sampled per cluster (herd).

The sample size calculation aimed to estimate the prevalence in each province with a precision, defined as one half-length of the 95% confidence interval, of 5%-points. We assumed seroprevalences of the different livestock species as were reported by Sukhbaatar in 2010 (3% for camels, 5% for goats, 7% for sheep, 8% for cattle). The calculated sample size for a province was to sample 30 herds each with at least 3 camels, 3 cattle, 5 sheep and 5 goats.

#### Selection of animals and sampling

In a selected herd, sheep and goats were selected when exiting an enclosure using the sampling interval i: total number of animals divided by 5. The first animal was selected with a random number and then every i^th^ sheep and goat was sampled. Camels and cattle were selected in the direction of the bottle head after the bottle was spun and a random number to tell which animals were to be included in that direction. Species, sex, age, breed, and main use for each animal were recorded on a data sheet, where any noted clinical symptoms (e.g. abortions) in the herd within the past months were also registered.

Blood samples were collected from the jugular vein using a Vacutainer® tube with disposable needle. Tubes were centrifuged for 5–10 min at 1000–1500 rpm, then serum was aliquoted into two 2 mL Eppendorf tubes®, which were stored on ice in a cool box and transported regularly to the Veterinary Laboratory at the Province Center, where they were kept at − 20 °C until transported to the School of Veterinary Medicine (SVM), Ulaanbaatar, and again stored at − 20 °C until further processing.

Vaginal swabs and/or milk samples for bacteriology were collected from individual animals with history of abortion. Swabs were placed in transport medium tube (BD BBL™Culture swab plus, Amies without Characoal, Becton Dickinson, France) and transported to the State Central Veterinary Laboratory (SCVL) in cool boxes. Milk samples consisted of 10–20 mL of milk taken from each teat. The first streams were discarded and then the milk sample was collected into a sterile vessel [44].

### Serological testing

All serum samples were tested by the Rose Bengal Test (RBT) using RBT antigen (Biocombinate, Mongolia). Camel and cattle sera were tested with a serum:RBT reactive ratio of 1:1 (25 μL 25 μL) and small ruminant sera were tested with a serum:RBT reactive ratio of 3:1 (75 μL, 25 μL), all for 4 min, as recommended by the World Organization for Animal Health [45] and according to Mongolian national standards [46]. Results were recorded as agglutination negative (−), doubtful (+/−), or positive (+, ++ or +++) according to the strength and time to reaction. All tests and readings were performed by the same person. The serological test results were transformed to a binary outcome with the cut-off of seropositivity set at positive + agglutination. The RBT test with camel sera performed with 99% specificity, which is comparable to other livestock, however, with a rather low sensitivity of 75% [47].

### Bacteriological examination

Milk samples were centrifuged to concentrate bacteria, at 6000–7000 g for 15 min in sealed tubes to avoid potential for aerosolization [7, 44]. A mixture of cream and deposit was streaked both on petri dishes with Farrell’s medium (*Brucella* medium base, CM0169; antibiotic supplement, SR0083, Oxoid™) and with CITA medium (blood agar base number 2, CM0271, Oxoid™; and antibiotic supplements vancomycin, colistin, nystatin, nitrofurantoin, amphotericin B, Sigma™, as well as containing 5–10%, inactivated horse serum, SR0035, Oxoid™). The inoculated plates were incubated at 37 °C in absence and presence of 10% CO_2_ for up to 2 weeks [44, 48]. A bacteriologist selected colonies based on *Brucella* colony morphology. These were stained by Gram stain (K001, Himedia) and modified Ziehl-Neelsen stain (21,820 Sigma™). In addition, reactivity to oxidase strips (MB0266A, Oxoid) was tested and both urea agar and urea broth were used for urease tests (urea agar 211,795, BD BBL™; Bacto agar 214,010 BD and Urease Test Broth 221,719, BBL™). Colonies positive for these tests were passaged to obtain pure cultures, from which DNA was extracted using G-Dex™llc Genomic DNA Extraction kit (iNtRON Biotechnology, Inc).

To identify *Brucella* species, the Bruce-Ladder multiplex PCR, using INgene Bruce-ladder (V R.10.BRU.k5) kits, was used. The PCR products were analyzed by 1.5% agarose gel electrophoresis (GelRed reagent used in place of Etidiumbromid, GelRed™ Nucleic Acid Gel Stain Biotium), and fragment sizes were estimated using the 1 kb plus DNA ladder as molecular size marker (Invitrogen). Gel images were captured with G-Box (G:Box F3 Syngene, USA).

### Questionnaires

The questionnaires were written in English and translated to Mongolian before pre-testing with 3 herder families in the vicinity of Ulaan Bator. The member of each selected camel-keeping family with the best knowledge on management of the camel herd was interviewed to obtain information about the herd, household and individual risk factors for brucellosis (Additional file 1). The interview included questions on i) knowledge of epidemiology of brucellosis ii) history of brucellosis in the household, iii) herd risk factors (including buying/selling of animals, sharing of pasture and watering places), iv) herd and human health management (including disposal of aborted fetuses/placentas), v) vaccination of cattle and small ruminants (Additional file 2). Questionnaires were not filled in on second visits to the same household. The coordinates of the household (*hot ail)* at time of the visit was recorded with a GPS. The mobile phone number of each participant was recorded for dissemination of results and to establish contact for second visits.

### Data management and analysis

Questionnaire and sample data were double entered in Microsoft Access® and compared and corrected using Epi-Info 3.5.3 (Centers for Disease Control and Prevention, USA). An identification system was used to uniquely identify all samples and individuals and facilitated merging data sets at province, district, household/herd and individual levels. Data analyses were done using Stata 14 (StataCorp IC, USA).

We calculated seroprevalences for brucellosis in camels using generalized estimating equations (GEE, Stata command xtgee) to account for clustering at herd level, which expands the confidence interval compared to simple binary confidence intervals (CI). The apparent seroprevelance was converted to an estimated true seroprevalence using the formula developed by Rogan and Gladen [49] to account for the fact that the apparent seroprevalence might be over- or underestimated. A multivariable GEE model accounting for clustering was used to assess the association of biologically plausible risk factors to the serological outcome. Since vaccination of other livestock was highly linked to province and year it was not included in the multivariable analysis. Exact logistic regression was used for explanatory variables, with zero cell counts in two-by-two tables. Other variables, such as knowledge of herders or preventive measures, were not tested as risk factors in the multivariable model in order to keep the model simple. Other variables were tested with univariable GEE models. Age categorization of camels was based on breeding maturity: young camels were ≤ 4 years and adult camels were >  4 years. The variance components at different sampling levels were determined with the generalised linear latent and mixed models (gllamm) command in Stata for hierarchical models. The ICC at the *hot ail* level was estimated with ANOVA. The ICC was estimated at the herd level because the variance components indicated that correlation within clusters was highest at this level, so it was used for the sample size calculation. Correlations between camel and other livestock herd seropositivity was done with linear regression models in R version 3.3.2. The 95% confidence intervals of the intercepts and slopes of the regressions were constructed using bootstrap re-sampling technique and the information on total number of livestock per herd and species.

We assigned scores to the questions on knowledge within three themes: transmission of brucellosis between herds, transmission from livestock to people, and clinical signs of livestock brucellosis. Correct answers were scored as 2, ‘Do not know’ as 0, and wrong answers as − 1. All scores within a knowledge theme were summed and the median taken to classify those with lower and higher scores.

## Additional files

Additional file 1: Hot ail questionnaire. The interview included Hot ail questions on herd risk factors (including buying/selling of animals, sharing of pastures and watering places), herd and human health management (including disposal of aborted fetuses/placentas), vaccination of cattle and small ruminants. (DOCX 29 kb)

Additional file 2: Herder questionnaire. The interview included questions on knowledge on epidemiology of brucellosis and history of brucellosis in the household. (DOCX 28 kb)

## Abbreviations

CI: Confidence Interval
GEE: Generalized estimating equation
ICC: Intraclass correlation coefficient
OR: Odds ratio
RBT: Rose Bengal Test
SCVL: State Central Veterinary Laboratory
SVM: School of Veterinary Medicine
