# Supplementary material for: Risk factors of brucellosis seropositivity in Bactrian camels of Mongolia
Source: BMC Vet Res. 2018 Nov 13;14:342. doi: 10.1186/s12917-018-1664-0 (PMC6234668; doi:10.1186/s12917-018-1664-0)
Supplement: Supplementary file 1 — Hot ail questionnaire. The interview included Hot ail questions on herd risk factors (including buying/selling of animals, sharing of pastures and watering places), herd and human health management (including disposal of aborted fetuses/placentas), vaccination of cattle and small ruminants. (DOCX 29 kb) [file 12917_2018_1664_MOESM1_ESM.docx]

**Hot ail (nomadic camp) questionnaire**

*Used for the survey on camel brucellosis in selected aimags (provinces) between 2013 and 2015. SDC Animal Health Project*

**Identification code:**

|__|__| |__¦__| |__¦__|

Aimag Soum Hot ail/hh

1. **Date of interview** |__|__|__|__| |__|__| |__|__|

Year Month Day

1. **Name of interviewer:** ………………………………………………………………….
2. **Coordinates of the hot ail:**

North I__I__I I__I__I__I__I__I__I, East I__I__I I__I__I__I__I__I__I

1. **Surname of the hot ail’s head**......................................... Name ………………………...
2. **Number of the households in hot ail** I___I___I

| N | Name of household head | Number of livestock | | | | | |
| --- | --- | --- | --- | --- | --- | --- | --- |
|  |  | Sheep | Goat | Horse | Cattle | Camel | Other |
| 1. |  |  |  |  |  |  |  |
| 2. |  |  |  |  |  |  |  |
| 3. |  |  |  |  |  |  |  |
| 4. |  |  |  |  |  |  |  |
| 5. |  |  |  |  |  |  |  |

1. **Has brucellosis been diagnosed in your livestock?**

|  | Species | Answer | | If yes, when (year) | Result | |
| --- | --- | --- | --- | --- | --- | --- |
|  |  | Yes | No |  | Positive,  how many? | Negative,  how many? |
| 1. | Cattle |  |  | I__I__I__I__I |  |  |
| 2. | Yak |  |  | I__I__I__I__I |  |  |
| 3. | Sheep |  |  | I__I__I__I__I |  |  |
| 4. | Goat |  |  | I__I__I__I__I |  |  |
| 5. | Camel |  |  | I__I__I__I__I |  |  |
| 6. | Horse |  |  | I__I__I__I__I |  |  |
|  | Total |  |  |  |  |  |

1. **Has your livestock been vaccinated during the past autumn?**

| N | Species | Answer | | If yes, when  (year, month) | Number of vaccinated livestock |
| --- | --- | --- | --- | --- | --- |
|  |  | Yes | No |  |  |
| 1. | Cattle |  |  | I__I__I__I__I. I__I__I |  |
| 2. | Yak |  |  | I__I__I__I__I. I__I__I |  |
| 3. | Sheep |  |  | I__I__I__I__I. I__I__I |  |
| 4. | Goat |  |  | I__I__I__I__I. I__I__I |  |

1. **Did you buy any animals the past 12 months?** Yes I__I No I__I

If yes (please fill in a table)

| 1. | From where |  |
| --- | --- | --- |
| 2. | When within last months | a). 1-3 b). 3-6 c). 6-12 d). .......... |
| 3. | Was that animal tested for brucellosis? |  |
| 4. | Was it noted in your herdbook? |  |

1. How many families share the pasture for their herds? I__I__I
2. How many families share the watering points for their livestock? I__I__I
3. How do you handle aborted material?

(please write) ………………………………………………………………………..

1. During the past calving season, did you have abortions in cattle Yes I__I No I__I
2. If yes, which trimester of the pregnancy? I__I__I
3. Did you observe swollen front knees and creaky noises of the joints in a camel?

Yes I__I No I__I

**Thank you very much for your participation**
