# Supplementary material for: Risk factors of brucellosis seropositivity in Bactrian camels of Mongolia
Source: BMC Vet Res. 2018 Nov 13;14:342. doi: 10.1186/s12917-018-1664-0 (PMC6234668; doi:10.1186/s12917-018-1664-0)
Supplement: Supplementary file 2 — Herder questionnaire. The interview included questions on knowledge on epidemiology of brucellosis and history of brucellosis in the household. (DOCX 28 kb) [file 12917_2018_1664_MOESM2_ESM.docx]

**Individual (herder) questionnaire**

*Used for the survey on camel brucellosis in selected aimags (provinces) between 2013 and 2015. SDC Animal Health Project*

Identification code:

|__|__| |__|__| |__¦__| |__|

Aimag Soum Hot ail/hh Herder id

Name of interviewer …………………………………..

**A. Information on the livestock owner**

1. **Date of the interview and blood sampling:** |__¦__¦__¦__| |__¦__| |__¦__|

Year Day Month

1. **Surname**………………………… **Name**…………………………………
2. **Date of birth** |__¦__¦__¦__| |__¦__| |__¦__|
3. **Sex** Male |__| Female |__|
4. **Occupation:** A: Herder |__| B: Other |__|
5. **Phone numbers**: |__|__|__|__|__|__|__|__| or: |__|__|__|__|__|__|__|__|
6. **Have you ever given blood to test for human brucellosis?** Yes |__| No |__|

If yes, when? |__|__|__|__| |__|__|

Year Month

Was the result positive? Yes |__| No |__|

**B: Knowledge on brucellosis**

1. **Which symptoms can brucellosis patients have?**

| A. Skin rash | yes \|__\| no \|__\| | G. Weakness | yes \|__\| no \|__\| |
| --- | --- | --- | --- |
| B. Fever | yes \|__\| no \|__\| | H. Night sweat | yes \|__\| no \|__\| |
| C. Arm and leg pain | yes \|__\| no \|__\| | I. Depression | yes \|__\| no \|__\| |
| D. Back pain | yes \|__\| no \|__\| | K. Abortion | yes \|__\| no \|__\| |
| E. Muscle pain | yes \|__\| no \|__\| | L. Testicle pain | yes \|__\| no \|__\| |
| F. Exhaustion | yes \|__\| no \|__\| | M. Headache | yes \|__\| no \|__\| |

1. **Which animals transmit brucellosis infection to human?**

|  | Yes | No |  | Yes | No |
| --- | --- | --- | --- | --- | --- |
| Cattle | I__I | I__I | Horse | I__I | I__I |
| Wolf | I__I | I__I | Dog | I__I | I__I |
| Goat | I__I | I__I | Sheep | I__I | I__I |
| Cat | I__I | I__I | Deer | I__I | I__I |
| Camel | I__I | I__I | Gazelle | I__I | I__I |

1. **Which of the following symptoms can animals infected with brucellosis show?**

A. Abortion Yes I__I No I__I

B. Delivery with difficulty Yes I__I No I__I

C. Weight loss Yes I__I No I__I

D. Lack of milk Yes I__I No I__I

E. Swollen leg joints Yes I__I No I__I

F. Limping for a long time Yes I__I No I__I

G. Animal tongue becomes blue Yes I__I No I__I

1. **How can a herd become infected with brucellosis?**

A. By mixing with a brucellosis infected herd I__I

B. By sharing the same pasture with a brucellosis infected herd I__I

C. By sharing watering places (well, river) with a brucellosis infected herd I__I

D. By introducing a single brucellosis infected animal into a herd I__I

1. **How can people become infected with brucellosis?**

A. By consuming raw milk I__I

B. By consuming raw milk products I__I

C. By consuming half-done meat I__I

D. By milking animals I__I

E. By contact with animal wool and skin I__I

F. By combing cashmere I__I

G. By shearing wool I__I

H. By contact with animal placenta I__I

I. By assisting in obstetric work I__I

1. **Do you use personal protective clothes during contact with animals?**

Yes I__I No I__I

If yes, what kind of personal protective clothes do you wear? (Please write)

....................................................................................................................................

…………………………..……..………………………………………..……………………..

1. **From where do you get information on brucellosis?**

A. From soum physicians and health care workers I__I

B. From veterinarian I__I

C. From promotion materials I__I

D. From radio and TV I__I

E. From newspapers and magazines I__I

F. From friends and relatives I__I

1. Do you have a traditional way of raw livestock processing?

Yes I__I No I__I

If yes, which products and how are they used? (*please describe*)

……………………………………………………………………………………………………….

1. Do you use personal protective clothes during the lambing season?

Yes I__I No I__I

If yes, which kind of personal protective clothes do you wear? (*please describe*)

…..……………………………………………………………………………………………………….

……………………………………………………………………………………………………………

1. Do you use disinfects for the livestock pen? And what else do you do?

(*please describe*)……………………………………………………………………………..…….

.……………………………………………………………………………………………………….

.……………………………………………………………………………………………………….

**Thank you very much for your participation**
